# Additive effects of social and non-social attention during infancy relate to later autism spectrum disorder

**DOI:** 10.1111/desc.12139

**Published:** 2014-02-23

**Authors:** Rachael Bedford, Andrew Pickles, Teodora Gliga, Mayada Elsabbagh, Tony Charman, Mark H Johnson

**Affiliations:** 1 Department of Biostatistics, Institute of Psychiatry, King's College LondonUK; 2 Centre for Brain and Cognitive Development, Birkbeck, University of LondonUK; 3 Department of Psychiatry, McGill UniversityCanada; 4 The BASIS team in alphabetical order: Simon Baron-Cohen, Patrick Bolton, Susie Chandler, Janice Fernandes Holly GarwoodKristelle Hudry, Greg Pasco, Leslie Tucker and Agnes Volein

## Abstract

Emerging findings from studies with infants at familial high risk for autism spectrum disorder (ASD), owing to an older sibling with a diagnosis, suggest that those who go on to develop ASD show early impairments in the processing of stimuli with both social and non-social content. Although ASD is defined by social-communication impairments and restricted and repetitive behaviours, the majority of cognitive theories of ASD posit a single underlying factor, which over development has secondary effects across domains. This is the first high-risk study to statistically differentiate theoretical models of the development of ASD in high-risk siblings using multiple risk factors. We examined the prediction of ASD outcome by attention to social and non-social stimuli: gaze following and attentional disengagement assessed at 13 months in low-risk controls and high-risk ASD infants (who were subsequently diagnosed with ASD at 3 years). When included in the same regression model, these 13-month measures independently predicted ASD outcome at 3 years of age. The data were best described by an additive model, suggesting that non-social attention, disengagement, and social attention as evidenced by gaze following, have a cumulative impact on ASD risk. These data argue against cognitive theories of ASD which propose that a single underlying factor has cascading effects across early development leading to an ASD outcome, and support multiple impairment models of ASD that are more consistent with recent genetic and neurobiological evidence.

## Introduction

Autism spectrum disorder (ASD) is defined behaviourally by a triad of impairments with difficulties in both social (social and communication difficulties) and more general functioning (restricted and repetitive behaviours) (ICD-10; World Health Organization, 1993). The majority of cognitive theories of ASD propose a single initial impairment in either social orienting or social information processing (e.g. social orienting, Dawson, Meltzoff, Osterling, Rinaldi & Brown, 1998; theory of mind, Baron-Cohen, Leslie & Frith, 1985) or a more domain general impairment, such as in attentional control (e.g. disengagement, Landry & Bryson, 2004). However, attempts to explain the whole range of behavioural symptoms seen in ASD based on any single core deficit have been unsuccessful (see Happé & Ronald, 2008).

The idea that ASD may have multiple causes has been around for several decades, with Wing and Wing (1971) arguing that ASD should be conceptualized in terms of a combination of impairments. More recently, cascading and cumulative risk models have been proposed to describe the role of multiple factors in the development of ASD (Elsabbagh & Johnson, 2010). In a cumulative risk model, multiple early risk factors act additively to exceed a particular threshold thus altering a child's developmental trajectory. Cascading effects models, on the other hand, imply interactions between different factors during development, while the brain is still highly plastic, leading to a non-linear profile of mapping from risk factors to outcome. Cumulative and cascading risk models make specific predictions in terms of the modularity or interaction between the brain systems subserving social and non-social cognition. Cumulative models predict summation, but little interaction between these systems over development, whereas cascading effects models predict the opposite pattern, with systems playing a central role in each other's specialization. While these models have been discussed with respect to atypical development (e.g. gene–environment models in Williams Syndrome; Karmiloff-Smith, 2006), there has been no formal empirical testing of these contrasting hypotheses in the early development of ASD.

One can only test the validity of cascading versus cumulative models using longitudinal data, which have only recently become available, from prospective studies of infants at-risk for ASD. Testing multivariate developmental theories of ASD also requires analysis exploiting statistical models that combine effects. Such models have been frequently used in epidemiology, and only recently have their theoretical applications to developmental psychopathology been described (Pickles, 1993; Pickles & De Stavola, 2007). If risk factors work through alternative pathways then the expectation of the simplest model predicting the rate of the outcome when both risk factors are present would be the sum of the increments when each was present singly – an additive model. By contrast, if risk factors worked on a common pathway a multiplicative effect would be expected. The routinely applied logistic regression model has many desirable statistical properties, but few users appreciate that in most applications where the pathological outcome is not the majority, this model has closer correspondence to the multiplicative combination of effects, which is more consistent with the cascade model than the cumulative risk model. In order to test cumulative effects, a non-logistic model is required, which treats the factors as additive.

We focus here on two abilities that have been associated with ASD but that have also been hypothesized to interact during development, gaze following and attentional disengagement. Various theories have proposed that social attention, such as joint attention (JA; i.e. jointly attending to an object with another person), is particularly, and specifically, impaired in ASD (e.g. Dawson *et al*., 1998). JA can be broken down into different components: disengagement from the face, reaction time and orienting to the object and looking time to the referred object. In both typical and atypical development several studies have shown looking time to be important in distinguishing infants’ understanding of the *meaning* of eye-gaze. From 10 months of age, infants show an increase in looking time to a target when an adult turns to an object with their eyes open versus closed (Brooks & Meltzoff, 2002, 2005). This increase in infants’ looking behaviour is an indication that they understand the importance of open eyes as a cue to the other person ‘seeing’ the object. This is consistent with our previous paper in which we found reduced looking time (but not first look responses) to a gazed-at object in infants who later developed symptoms of ASD compared to low-risk and other high-risk infants (Bedford, Elsabbagh, Senju, Gliga, Pickles, Charman, Johnson & the BASIS Team, 2012).

Emerging findings from high-risk studies document a range of JA-type difficulties from the first year of age. Presmanes, Walden, Stone and Yoder (2007) found reduced looking to the target in 12- to 23-month-old high-risk children compared to low-risk controls when given an intermediate number of cues to follow (e.g. gaze shift + vocalization) rather than a single cue or a highly redundant combination of multiple cues. Response to JA at 14 and 15 months has also been shown to be a significant predictor of ASD outcome (e.g. Sullivan, Finelli, Marvin, Garrett-Mayer, Bauman & Landa, 2007; Yoder, Stone, Walden & Malesa, 2009; Rozga, Hutman, Young, Rogers, Ozonoff, Dapretto & Sigman, 2011).

Other high-risk studies have examined early disengagement of visual attention using the gap-overlap task. In this task a central stimulus is followed by the appearance of a peripheral stimulus either: (1) simultaneously with the offset of the central stimulus (baseline); or (2) while the central stimulus is still on the screen (overlap). Disengagement is the difference in reaction time between the overlap and baseline conditions. Elsabbagh, Volein, Holmboe, Tucker, Csibra, Baron-Cohen, Bolton, Charman, Baird and Johnson (2009) found increased disengagement latencies in the high-risk as compared to low-risk infants at 9–10 months of age. Similarly, Zwaigenbaum, Bryson, Rogers, Roberts, Brian and Szatmari (2005) found that a decrease in disengagement ability between 6 and 12 months predicted ASD as measured by the ADOS-G at 24 months. Twenty-five percent of their high-risk sample showed this pattern of increased latency to disengage at 12 months of age compared with performance at the earlier 6-month assessment. *All* of the infants in this subgroup went on to receive a diagnosis of ASD on the ADOS-G, which suggests that this factor on its own may be a strong predictor of ASD.

JA abilities change across the first year of life, from a preference for direct gaze and faster orienting given gaze cues in neonates (Farroni, Csibra, Simion & Johnson 2002; Farroni, Massaccesi, Pividori & Johnson, 2004) to the development of more flexible JA behaviours towards the end of the first year of life (Butterworth & Jarrett, 1991; Scaife & Bruner, 1975). One proposed source of developmental change is changes in attention control, the ability to flexibly disengage and shift attention in a visual scene. In order to engage in JA, it is necessary to disengage from distracting objects and look at a person's face and then again switch attention to look at the gazed-at object. It is therefore plausible that early difficulties with flexibly switching attention lead to problems in social gaze following. Although Leekam, López and Moore (2000) showed a dissociation, with relatively intact disengagement of attention but difficulties in JA, in young preschool children with ASD, it may be that these abilities interact earlier during development.

However, in order to test the relationship between social and non-social attention in relation to ASD outcome, a prospective longitudinal design is required. We have previously reported difficulties for 13-month-old infants who later developed ASD in both gaze following behaviour (Bedford *et al*., 2012) with reduced looking time to the congruent (gazed-at) object, and attentional flexibility (Elsabbagh, Fernandes, Webb, Dawson, Charman, Johnson & the BASIS Team, 2013), with longer disengagement latencies in a gap-overlap task. In both of these studies, gaze following and disengagement were also measured at 7 months, but at this age there were no group differences in relation to ASD outcome. The focus of the current paper is to use the data from these previously published studies (Bedford *et al*., 2012; Elsabbagh *et al*., 2013) to test theoretical models of ASD development. More specifically, while we already know that both 13-month gaze following behaviour and attentional disengagement relate to ASD, the aim of the present study is to test whether these measures work *together*, and whether they act additively or multiplicatively to predict ASD outcome.

## Method

### Participants

#### Gap-overlap task: 13 months (see Elsabbagh *et al*., 2013)

At 13 months, 45 low-risk controls, 22 high-risk (HR)-typical and 17 HR-ASD infants took part in the gap-overlap task (see Table1). Four low-risk infants were excluded owing to non-compliance or technical difficulties.

**Table 1:** Descriptive statistics for the Mullen Scales of Early Learning (MSEL) Early Learning Composite Standard Scores (ELC) at 13 and 36 months, and disengagement and looking time scores at 13 months

	Low-risk controls	*‘HR-ASD’*	*‘HR-Typical’*
M (SD)	M (SD)	M (SD)
Attentional Disengagement/ms	137.52 (106.91)	268.82 (157.62)	137.34 (135.84)
Valid trials	27.36 (6.65)	24.41 (6.53)	24.86 (7.54)
	N = 45	N=17	N=22
Proportion Looking Time	0.31 (0.14)	0.22 (0.08)	0.30 (0.11)
Valid trials	5.51 (2.16)	5.64 (2.06)	5.3 (2.32)
	N = 37	N=ll	N=13
13m*MSEZ* ELC	106.41 (15.76)	89.18 (18.30)	103.35 (18.12)
	N = 46	N=17	N=23
36m*MSEL* ELC	115.77 (16.25)	94.75 (28.51)	113.46 (13.26)
	N = 48	N=16	N=24

Gaze following task: 13 months (see Bedford *et al*., 2012

The same 37 low-risk, 13 HR-typical and 11 HR-ASD from the Bedford *et al*. (2012) study were included in the analysis. Bedford *et al*.'s (2012) inclusion criteria required infants to have data on the task at both 7 and 13 months. Thus, while here only the 13-month data (which was the time point that related to later ASD diagnosis) are analysed, the same subsample was used to allow direct comparison across papers.

### Procedure

#### Gap-overlap task

The task and procedure are identical to that used by Elsabbagh *et al*. (2013) with infants seated 60 cm from the computer screen on their parent's lap and the stimuli were presented on a 46″ LCD monitor. Looking behaviour was recorded using a video camera. The rate of trial presentation was controlled by the experimenter. Each trial began with a centrally presented animation subtending 13.8° × 18.0°, which rotated to attract the infant's attention. Once the infant looked to the centre of the screen a peripheral target, subtending 6.3° × 6.3°, appeared randomly on either the left or right of the screen (eccentricity of 15.0°). The peripheral targets were green balloons that expanded and contracted to get the infant's attention. The target remained on the screen until either (1) the infant looked at it; or (2) the maximum time limit of 2.5 seconds passed. When one of these criteria had been fulfilled, an animal (elephant, lion, seal, etc.) appeared accompanied by a sound, replacing the target green balloon.

Infants were presented with a maximum of 70 trials, but the task was stopped sooner if they became fussy. There were two different trial types: baseline and overlap. In the baseline condition, the central stimulus disappeared at the *same time* as the peripheral target appeared, whereas in the overlap condition, the central stimulus remained present (but not moving) while the target stimulus appeared in the periphery. The conditions were presented pseudo-randomly across two blocks (one with a sun and the other with a clown as the central stimulus).

#### Gaze following task

The procedure and stimuli used are identical to those used by Bedford *et al*. (2012). Infants were seated on their parent's lap at a distance of 50 cm from the screen and looking behaviour was recorded using a Tobii 1750 eye-tracker. The eye-tracker has an infrared light source and a camera mounted below a 17″ flat screen monitor to record corneal reflection data. To evaluate where on the screen the infant is looking, the Tobii system used measurements of gaze direction from each eye separately. Stimuli were presented on the screen using ClearView software. Before starting the main experimental task, a 5-point calibration sequence was run and the task was started when at least 4 points were marked as correctly calibrated for each eye. Gaze data were recorded at 50 Hz, and the spatial resolution was 1° after calibration.

Before the beginning of each trial, the infant's attention was directed to the screen using small animations. The trials began with two objects on a table and a female model ‘looking down’ (3 seconds), then looking up – ‘direct gaze’ (2 seconds) – and then turning her head to look at one of the objects – ‘shift’ (6 seconds). The ‘looking down’ phase was measured from the start of the trial until the model looked up and both her head and eye-gaze were directed straight ahead. The ‘direct gaze’ phase began as soon as the model's eyes were looking ahead, and finished when her head began to turn away. This turning marked the beginning of the ‘shift’ phase, which finished at the end of the trial. The object looked at by the model during ‘shift’ is the *congruent* object, and the other, non-gazed at object is the *incongruent* object. Each infant viewed 12 trials, and there were six different pairs of objects whose position with respect to the gaze was counterbalanced across trials.

### Analysis and measures

#### Gap-overlap task

In the gap-overlap task, data were video coded frame-by-frame by two raters, who established a reliability of > 0.9 (Cohen's K) for trial validity and a correlation of 0.87 for saccadic reaction time on a practice data set. There was no difference in the number of valid trials completed by low-risk infants and HR-ASD, *t*(60) = 1.56, *p* = .12, or HR-typical and HR-ASD, *t*(37) = 0.2, *p* = .85. Invalid trials were those in which the infant (1) looked away from the screen; (2) did not look at the central stimulus immediately before the onset of the peripheral stimulus; (3) looked away or blinked during the onset of the peripheral stimulus.

*Disengagement*. This measure of disengagement comes from the previously published paper by Elsabbagh *et al*. (2013) with the same cohort of children, and has been used for over three decades (see Fischer, Gezeck & Hartnegg, 1997; Posner, 1980). Saccadic reaction times were analysed from all valid trials in which the infants oriented towards the peripheral stimulus 100ms–1200ms after its onset. If the infant did not look towards the stimulus in this time the trial was called ‘failure to disengage’ and reaction time was not analysed. Because reaction time cannot be calculated when infants fail to disengage, these trials were not analysed for the purpose of this paper. Elsabbagh *et al*. (2009, 2013) analysed these trials separately as a percentage of total number completed and found no significant group differences. A measure of ‘disengagement’ was calculated as reaction time in overlap trials − baseline trials. This measure of attentional disengagement was chosen because, as discussed earlier, disengagement of attention has been argued to underlie the development of gaze following behaviour.

#### Gaze following task

Trial exclusion criteria were: (1) no looking to the face during ‘direct gaze’ as Senju and Csibra (2008) found this to be a prerequisite for gaze following behaviour; and (2) looking away from the computer screen for the entire ‘shift’ phase. Only data from the final ‘shift’ phase were used to calculate the measures of interest.

#### Looking time

This measure is taken from Bedford *et al*. (2012) using the same cohort of children. Looking time behaviour was analysed only for trials in which infants had a correct first look to the congruent object; three infants were excluded as they had no correct first looks (1 low-risk, 1 HR-typical, 1 HR-ASD). There were no significant group differences in total looking time at 13 months either between low-risk and HR-ASD, *t*(46) = −0.70, *p* = .49, or HR-typical and HR-ASD, *t*(22) = −0.58, *p* = .57. Looking time to the congruent object (out of total looking time to the slide) during the ‘shift’ phase was calculated for all first look trials that were correct. Only correct trials were analysed because we were specifically interested in whether, having made a correct first look, infants behaved differently in their looking to the object. This measure reflects not only the infants’ ability to follow gaze but also their subsequent engagement with the target of another person's gaze.

## Behavioural assessments and clinical classification

For the high-risk group consensus ICD-10 (World Health Organization, 1993) *ASD diagnoses* (HR-ASD; childhood autism; atypical autism; other pervasive developmental disorder; PDD) were achieved using all available clinical information from all visits by experienced researchers (TC, KH, SC, GP). Given the young age of the children, and in line with the changes to DSM-5, no attempt was made to assign specific sub-categories of PDD/ASD diagnosis. Toddlers from the high-risk group were considered typically developing (HR-typical) at 36 months if they (1) did not meet ICD-10 criteria for an ASD; (2) did not score above the cut-off on the ADOS-G or ADI-R; (3) scored within 1.5 *SD* of the population mean on the MSEL Early Learning Composite (ELC) (Mullen, 1995) standard score (> 77.5) and RL and EL subscale T-scores (> 35). Finally, toddlers from the high-risk group were considered to have other developmental concerns if they did not fall into either of the above groups. However, for the purposes of the current paper, only high-risk ‘ASD’ and ‘typical’ children were included in the analysis. From the 53 (out of 54) high-risk infants seen for diagnostic assessment at 36 months, 17 (11 male, 6 female) were classified as HR-ASD (32.1%), 24 (7 male, 17 female) as HR-typical (45.3%) (see Table S1). While our recurrence rate of ASD is higher than the recently reported rates of ∼20% (Ozonoff, Young, Carter, Messinger, Yirmiya, Zwaigenbaum, Bryson, Carver, Constantino, Dobkins, Hutman, Iverson, Landa, Rogers, Sigman & Stone, 2011) this is likely due to variability resulting from our relatively modest sample.

## Statistical analysis

There are good statistical reasons for the popularity of the logistic regression model, an example of a generalized linear model (GLM) with binomial error and logit link function. The simple additive model (a GLM with Gaussian error and identity link function) can yield predictions outside the feasible range of 0 to 1, while the simple multiplicative (a GLM with Gaussian error and log link) can yield predictions beyond 1. Further, for both multiplicative and additive models the relationship between the residual variance and the predicted proportion, which is implicitly accounted for in the binomial error of the logit model, must be accounted for to obtain correct standard errors. In fitting explicitly additive and multiplicative models we therefore checked for predictions beyond the feasible range and implemented in the Stata procedure gllamm (Rabe-Hesketh, Skrondal & Pickles, 2004), an iterative estimation method in which the residual variance was made a linear function of the predicted proportion. Since in this implementation the additive and multiplicative models are both Gaussian, their relative fit as reflected in the respective log-likelihood values can be directly compared.

## Results

No significant correlations between disengagement (gap-overlap task) and looking time (gaze following task) were found at 13 months for either the low-risk controls (*r* = −0.02, *p* = .90) or the HR-ASD group (*r* = −0.12, *p* = .74). The two measures were thus entered as separate predictors in the following models. Thirteen-month MSEL was initially entered into the model as a covariate, but it was dropped because it was not significant (*p* = .12).

### Prediction of clinical outcome by 13-month measures

Disengagement scores were divided by 1000 (i.e. converted from milliseconds to seconds) to ensure that both variables were on a similar scale. The low-risk controls were the baseline group and were compared to the ASD outcome group.

**Figure 1:**
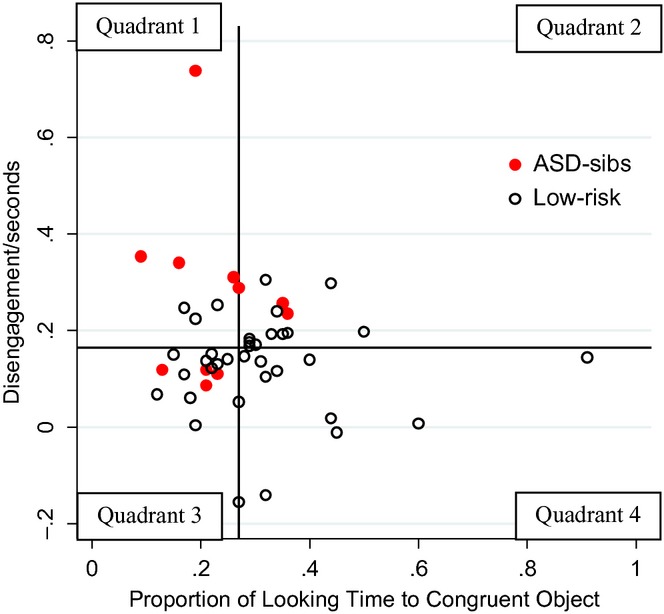
Scatterplot of looking time and disengagement scores split by HR-ASD versus low-risk control outcome. Lines represent median values, and separate the data into four quadrants.

A scatterplot (see Figure1) indicates that some children with ASD show a difficulty in either disengagement (i.e. increased saccadic RTs) or looking time (reduced looking to the congruent object), with others having problems with both behaviours. None of the children with ASD fall in quadrant 4, with difficulties in neither of the behaviours.

To demonstrate that the multiplicative model is acting in much the same way as the logistic model, predicted values were generated for a child from each quadrant. The predicted probability for the selected child in quadrant 1 (i.e. with slow disengagement and reduced looking time, both risk factors) having ASD is 0.3 under an additive model, whereas the values from the logistic and multiplicative models were much higher, 0.82 and 0.62, respectively (see Table2). Thus, while all three models give similar predictions for quadrants 2–4 (children who show either no impairments or difficulties with only one of the two behaviours), for those children with difficulties in both gaze following and disengagement, the additive model makes different predictions to the multiplicative and logistic. In the following analysis, results from a multiplicative and an additive model, with looking time and disengagement as the two predictors of ASD outcome, were compared.

**Table 2:** Observed disengagement and looking time scores for four infants, one from each quadrant of Figure1, and the associated predicted probabilities under logistic, multiplicative and additive models

Quadrant	Scores		Predicted probabilities		
	Disengagement/s	Proportion of looking time	Logistic	Multiplicative	Additive
1	0.34	0.16	0.82	0.62	0.30
2	0.23	0.36	0.18	0.18	0.19
3	0.09	0.21	0.14	0.20	0.14
4	0.02	0.44	0.01	0.06	0.04

**Figure 2:**
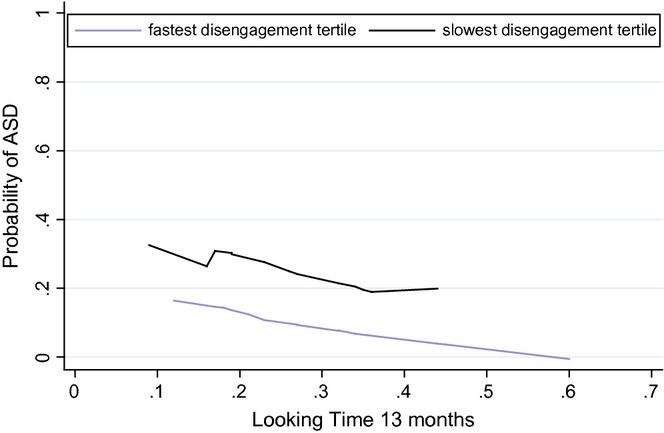
Additive model showing the effect of 13-month looking time on ASD outcome for fastest and slowest disengagement tertiles.

The additive model (loglikelihood = −14.18) provided a better fit to the data than the multiplicative model (loglikelihood = −19.77), and showed both looking time (coef = 0.271, *SE* = 0.119, *z* = −2.28, *p* = .023) and disengagement (coef = 0.549, *SE* = 0.227, *z* = 2.41, *p* = .016) to be significant predictors of ASD outcome. Under the additive model children who have reduced looking time to the congruent object and high disengagement scores at 13 months were more likely to classified as HR-ASD versus low-risk control. A lowess smooth fitted to the predicted probabilities for the lowest and highest tertile of disengagement (Figure2) shows two parallel lines indicative of the additivity of effects under this model. Those infants with both reduced looking time in the gaze following task and slow disengagement (black line) have a higher chance of an ASD outcome than those with faster disengagement (purple line). However, this increased risk is close to the sum of each individual risk, an additive effect. Under the multiplicative model neither of the factors is a significant predictor.

Although we have no reason to think the two potential ‘outliers’ (see Figure1) reflect anything other than variability in a relatively modest sample, we repeated the analysis excluding the two points and found substantively similar results, with the additive model remaining the significantly better fitting model (additive LL = −14.84, multiplicative LL = −19.90). Under the additive model disengagement remained as a significant predictor of ASD outcome (*p* = .03) while looking time tended to significance (*p* = .09).

We also repeated the analysis comparing HR-ASD to HR-typical infants (who showed no symptoms of ASD on ADOS-G or ADI-R and were within normal range on the MSEL; see Table S1). While the additive model provided a numerically better fit (additive LL −13.65 vs. multiplicative LL −14.05), the difference was not significant, potentially due to the decreased sample size. Under the additive model both attentional disengagement and looking time became marginal predictors (*p* = .05 and .09, respectively).

## Discussion

Understanding how biological risk factors combine in the development of psychopathology is of etiological importance and may in the longer term have implications for prognosis. This is the first study to formally test between single versus multiple factor accounts of ASD using developmental data. Using attentional disengagement and gaze following at 13 months, an additive model provided the best fit to the data and showed that both measures independently predicted ASD outcome. These results suggest that measures of attention taken both in a social and a non-social context contribute to ASD outcome via separate pathways, a finding which is in contrast to the predictions of the majority of cognitive theories of ASD which usually propose a single underlying pathway.

Both 13-month ‘looking time’ from the gaze following task and ‘disengagement’ from the gap-overlap task remained significant predictors of ASD outcome versus low-risk controls when entered into the same additive regression model. A very similar pattern was found for ASD infants versus those high-risk infants with a typical outcome, although the predictors did not reach significance, likely due to the reduced sample size.

As Figure1 indicates, several children who go on to an ASD diagnosis in this sample exhibit difficulties in only one of the two measured behaviours, i.e. in either social or non-social attention, consistent with effects combining in a sub-multiplicative fashion. This independent prediction of ASD outcome is similar to the findings of a recent study which found separate contributions from multiple factors (executive functioning, reaction time and emotional functioning) in the prediction of ADHD (Sjöwall, Roth, Lindqvist & Thorell, 2012). Further, the better fit of the additive model is more in line with the predictions of cumulative risk models, which propose that factors work in an additive way to increase risk for ASD. Figure2 shows the clear linear relationship between looking time and ASD outcome for both high and low disengagement reaction times.

Whether these two factors converge in affecting the same type of symptoms, or result in different symptoms of ASD, is something for future studies to investigate using larger samples. The ‘fractionable triad hypothesis’ (Happé & Ronald, 2008) argues that social and non-social symptoms are separable all the way from genes to the clinical phenotype. Recent neurobiological accounts have emphasized the heterogeneity of underlying mechanisms in the development of ASD (see Abrahams & Geschwind, 2008, for a review). Similarly, Geschwind (2009) proposes that there are in fact multiple ‘autisms’ with distinct etiologies, although he argues that different genetic routes map onto common cognitive phenotypes. While the independent relationship between looking time and disengagement with clinical outcome supports the idea of separate risk factors in ASD development, the fractionable triad view might go further and predict that early social attention should map more closely onto social-communicative symptoms and non-social attention onto restricted and repetitive behaviours (RRBIs). Thus, we would expect looking time and disengagement to predict different symptoms but in the same individuals. This link with phenotypic variability has not been analysed in the current study due to the limited size of the ASD outcome group (*N* = 17), but the question of individual differences is important. It may also be that the subgroup of children who show difficulties in both domains are qualitatively different from those who have impairments in only one domain, i.e. they may have earlier onset ASD, or greater symptom severity. These are questions which should be addressed in future by more highly powered studies.

This study analysed the combined effect of looking time (from a gaze following task) and attentional disengagement (from a gap-overlap task) in relation to ASD outcome. Applying statistical models to developmental data in order to test theoretical models is a crucial step in understanding the underlying mechanisms in the emergence of ASD. While this study is the first to examine the effect of multiple risk factors in the prediction of ASD outcome, we acknowledge that only two measures from a single time point were analysed. Future studies should build on this and establish how multiple cross-domain risk factors relate to one another, and to later ASD development, across the first few years of life.

In conclusion, at 13 months, both looking time to the congruent object and attentional disengagement predict ASD outcome in an additive manner, with high-risk infants who show reduced looking time to the object, and slower disengagement, more likely to develop ASD. These findings are not compatible with the majority of cognitive theories of ASD, which propose one primary causal factor. The results are in line with the cumulative risk account of ASD development, although it is also possible that different factors may relate to different phenotypic profiles, as proposed by the fractionable triad hypothesis. Understanding the developmental origin of phenotypic heterogeneity in ASD will be an important step in the development of targeted intervention strategies.

## Supporting Information

Data S1 Participants and Clinical Characterization.
